# Crystal structure of canagliflozin hemihydrate

**DOI:** 10.1107/S2056989016006769

**Published:** 2016-04-26

**Authors:** Kai-Hang Liu, Jian-Ming Gu, Xiu-Rong Hu, Gu-Ping Tang

**Affiliations:** a Chemistry Department, Zhejiang University, Hangzhou, Zhejiang 310028, People’s Republic of China; b Center of Analysis and Measurement, Zhejiang University, Hangzhou, Zhejiang 310028, People’s Republic of China

**Keywords:** crystal structure, canagliflozin, hydrogen bonding

## Abstract

There are two canagliflozin mol­ecules (*A* and *B*) and one water mol­ecule in the asymmetric unit of the title compound, C_24_H_25_FO_5_S·0.5H_2_O [systematic name: (2*S*,3*R*,4*R*,5*S*,6*R*)-2-(3-{[5-(4-fluoro­phen­yl)thio­phen-2-yl]meth­yl}-4-methylphen­yl)-6-(hy­droxy­meth­yl)-3,4,5,6-tetra­hydro-2*H*-pyran-3,4,5-triol hemihydrate]. The dihedral angles between the methyl­benzene and thio­phene rings are 115.7 (4) and 111.7 (4)°, while the dihedral angles between the fluoro­benzene and thio­phene rings are 24.2 (6) and 20.5 (9)° in mol­ecules *A* and *B*, respectively. The hydro­pyran ring exhibits a chair conformation in both canagliflozin mol­ecules. In the crystal, the canagliflozin mol­ecules and lattice water mol­ecules are connected *via* O—H⋯O hydrogen bonds into a three-dimensional supra­molecular architecture.

## Abstract 2

In canagliflozin hemihydrate, the hydro­pyran ring exhibits a chair conformation in both canagliflozin mol­ecules. In the crystal, the canagliflozin mol­ecules and lattice water mol­ecules are connected *via* O—H⋯O hydrogen bonds into a three-dimensional supra­molecular architecture.

## Chemical context   

Canagliflozin is a member of a new class of anti-diabetic drugs which are used to improve glycemic control of diabetics (Cefalu *et al.*, 2013 ▸). The crystalline forms of canagliflozin have been reported (Mitsubishi *et al.*, 2013 ▸; Ahmed *et al.*, 2013 ▸; Chen *et al.*, 2013 ▸), we report here the single-crystal structure of the title compound.
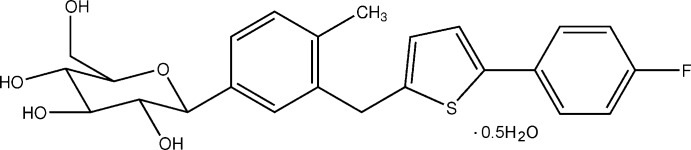


## Structural commentary   

**Figure 1:**
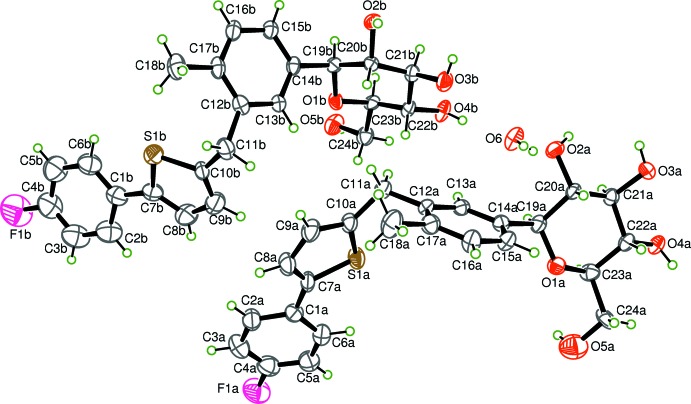
The mol­ecular structure of the title compound, (I), showing the atom-labeling scheme and displacement ellipsoids at the 40% probability level. H atoms are shown as small circles of arbitrary radii.

The title compound crystallizes with two independent canagliflozin mol­ecules and one water mol­ecule in the asymmetric unit (Fig. 1 ▸). The water mol­ecule links the two canagliflozin mol­ecules (*A* and *B*) *via* two O—H⋯O hydrogen bonds (Table 1 ▸).

The conformations of the two canagliflozin mol­ecules are somewhat different with regard to the orientation of the central benzene ring (C12–C17) with respect to the thio­phene ring, as indicated by torsion angles C9*A*—C10*A*—C11*A*—C12*A* = 113.3 (6)° in mol­ecule *A* and C9*B*—C10*B*—C11*B*—C12*B* = 108.0 (6)° in mol­ecule *B*. The conformational difference is also shown by the angle C10—C11—C12, which is 115.7 (4)° in mol­ecule *A* and 111.7 (4)° in mol­ecule *B*. The terminal aromatic rings (C1–C6) are inclined to the thio­phene rings, forming dihedral angles of 24.2 (6) and 20.5 (9)° in mol­ecules *A* and *B*, respectively. The tetra­hydro­pyran rings exhibit a distorted chair conformation in both mol­ecules *A* and *B*.

## Supra­molecular features   

**Figure 2:**
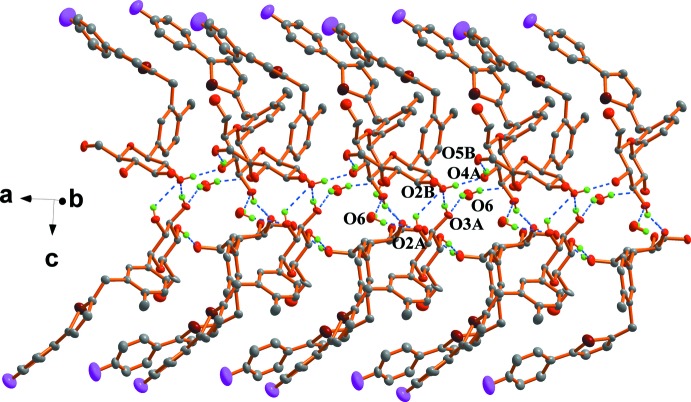
Part of the crystal packing of the title compound, showing the extensive inter­molecular hydrogen-bonding inter­actions (dashed lines). H atoms not involved in hydrogen bonding have been omitted for clarity.

In the crystal, O3*B*—H3*B*1⋯O4*B* ^i^, O2*B*–H2*B*1⋯O4*A* ^iii^, and O5*B*—H5*B*1⋯O3*B* ^iv^ [symmetry code: (i) *x* − 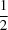, −*y* + 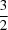, −*z* + 1; (iii) *x*, *y* + 1, *z*; (iv) *x* + 1, *y*, *z*] link canagliflozin mol­ecules, generating a ring of graph-set motif 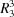
(9). The presence of the water mol­ecules results in the formation of zigzag chains mediated by alternating O4*B*—H4*B*⋯O6, O6—H61⋯O2*A* and O4*A*—H4*A*⋯O5*B* ^ii^ [symmetry code: (ii) *x* − 1, *y* − 1, *z*] hydrogen bonds propagating along the *a* axis; the chains are stacked along the *c* axis by further hydrogen-bonding inter­actions, O3*A*—H3*A*1⋯O2*B* ^i^ and O2*A–*-H2*A*1⋯O2*B* ^i^ (Fig. 2 ▸).

## Synthesis and crystallization   

The crude product was supplied by Zhejiang Huadong Pharmaceutical Co., Ltd. It was recrystallized from methanol solution, giving colorless crystals suitable for X-ray diffraction.

## Refinement   

Crystal data, data collection and structure refinement details are summarized in Table 2 ▸. All H atoms were placed in calculated positions with C—H = 0.93–0.98 Å and O—H = 0.82 Å and included in the refinement using a riding model, with *U*_iso_(H) = 1.2*U*_eq_ or 1.5*U*_eq_(carrier atom).

## Supplementary Material

Crystal structure: contains datablock(s) I, global. DOI: 10.1107/S2056989016006769/xu5886sup1.cif

Structure factors: contains datablock(s) I. DOI: 10.1107/S2056989016006769/xu5886Isup2.hkl

Supporting information file. DOI: 10.1107/S2056989016006769/xu5886Isup3.cml

CCDC reference: 1475516

Additional supporting information:  crystallographic information; 3D view; checkCIF report

## Figures and Tables

**Table 1:** Hydrogen-bond geometry (Å, °)

*D*—H⋯*A*	*D*—H	H⋯*A*	*D*⋯*A*	*D*—H⋯*A*
O2*A*—H2*A*1⋯O2*B* ^i^	0.82	2.42	2.841 (4)	113
O3*A*—H3*A*1⋯O2*B* ^i^	0.82	2.17	2.951 (4)	158
O4*A*—H4*A*⋯O5*B* ^ii^	0.82	1.98	2.756 (5)	157
O2*B*—H2*B*1⋯O4*A* ^iii^	0.82	1.85	2.672 (4)	179
O3*B*—H3*B*1⋯O4*B* ^i^	0.82	1.99	2.797 (4)	168
O4*B*—H4*B*⋯O6	0.82	1.93	2.749 (5)	172
O5*B*—H5*B*1⋯O3*B* ^iv^	0.82	2.31	3.015 (5)	144
O6—H61⋯O2*A*	0.82	2.23	3.031 (5)	166
O6—H62⋯O3*A* ^v^	0.83	2.30	3.058 (5)	153

Symmetry codes: (i) 
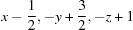
; (ii) 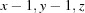; (iii) 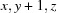; (iv) 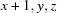; (v) 
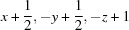
.

**Table 2:** Experimental details

Crystal data	
Chemical formula	2C_24_H_25_FO_5_S·H_2_O
*M*_r_	907.02
Crystal system, space group	Orthorhombic, *P*2_1_2_1_2_1_
Temperature (K)	296
*a*, *b*, *c* (Å)	8.4259 (4), 11.4264 (7), 45.706 (2)
*V* (Å^3^)	4400.4 (4)
*Z*	4
Radiation type	Mo *K*α
μ (mm^−1^)	0.19
Crystal size (mm)	0.48 × 0.28 × 0.26

Data collection	
Diffractometer	Rigaku R-AXIS RAPID
Absorption correction	Multi-scan (*ABSCOR*; Higashi, 1995 ▸)
*T*_min_, *T*_max_	0.914, 0.952
No. of measured, independent and observed [*I* > 2σ(*I*)] reflections	43211, 9958, 5079
*R*_int_	0.145
(sin θ/λ)_max_ (Å^−1^)	0.649

Refinement	
*R*[*F* ^2^ > 2σ(*F* ^2^)], *wR*(*F* ^2^), *S*	0.080, 0.147, 1.00
No. of reflections	9958
No. of parameters	575
H-atom treatment	H-atom parameters constrained
Δρ_max_, Δρ_min_ (e Å^−3^)	0.38, −0.29
Absolute structure	Flack (1983 ▸), 3246 Friedel pairs
Absolute structure parameter	0.13 (11)

Computer programs: *PROCESS-AUTO* (Rigaku, 2006 ▸), *CrystalStructure* (Rigaku, 2007 ▸), *SHELXS97* and *SHELXL97* (Sheldrick, 2008 ▸), *ORTEP-3 for Windows* and *WinGX* (Farrugia, 2012 ▸) and *DIAMOND* (Brandenburg & Putz, 2005 ▸).

## supplementary crystallographic information

### Crystal data

**Table:**

2C_24_H_25_FO_5_S·H_2_O	*F*(000) = 1912
*M**_r_* = 907.02	*D*_x_ = 1.369 Mg m^−^^3^
Orthorhombic, *P*2_1_2_1_2_1_	Mo *K*α radiation, λ = 0.71073 Å
Hall symbol: P 2ac 2ab	Cell parameters from 23292 reflections
*a* = 8.4259 (4) Å	θ = 3.0–27.4°
*b* = 11.4264 (7) Å	µ = 0.19 mm^−^^1^
*c* = 45.706 (2) Å	*T* = 296 K
*V* = 4400.4 (4) Å^3^	Needle, colorless
*Z* = 4	0.48 × 0.28 × 0.26 mm

### Data collection

**Table:**

Rigaku R-AXIS RAPID diffractometer	9958 independent reflections
Radiation source: rotating anode	5079 reflections with *I* > 2σ(*I*)
Graphite monochromator	*R*_int_ = 0.145
Detector resolution: 10.00 pixels mm^-1^	θ_max_ = 27.5°, θ_min_ = 3.0°
ω scans	*h* = −10→10
Absorption correction: multi-scan (*ABSCOR*; Higashi, 1995)	*k* = −14→14
*T*_min_ = 0.914, *T*_max_ = 0.952	*l* = −59→59
43211 measured reflections	

### Refinement

**Table:**

Refinement on *F*^2^	Secondary atom site location: difference Fourier map
Least-squares matrix: full	Hydrogen site location: inferred from neighbouring sites
*R*[*F*^2^ > 2σ(*F*^2^)] = 0.080	H-atom parameters constrained
*wR*(*F*^2^) = 0.147	*w* = 1/[σ^2^(*F*_o_^2^) + (0.0408*P*)^2^ + 2.8647*P*] where *P* = (*F*_o_^2^ + 2*F*_c_^2^)/3
*S* = 1.00	(Δ/σ)_max_ < 0.001
9958 reflections	Δρ_max_ = 0.38 e Å^−^^3^
575 parameters	Δρ_min_ = −0.29 e Å^−^^3^
0 restraints	Absolute structure: Flack (1983), 3246 Friedel pairs
Primary atom site location: structure-invariant direct methods	Absolute structure parameter: 0.13 (11)

### Special details

**Table:**

Geometry. All esds (except the esd in the dihedral angle between two l.s. planes) are estimated using the full covariance matrix. The cell esds are taken into account individually in the estimation of esds in distances, angles and torsion angles; correlations between esds in cell parameters are only used when they are defined by crystal symmetry. An approximate (isotropic) treatment of cell esds is used for estimating esds involving l.s. planes.
Refinement. Refinement of F^2^ against ALL reflections. The weighted R-factor wR and goodness of fit S are based on F^2^, conventional R-factors R are based on F, with F set to zero for negative F^2^. The threshold expression of F^2^ > 2sigma(F^2^) is used only for calculating R-factors(gt) etc. and is not relevant to the choice of reflections for refinement. R-factors based on F^2^ are statistically about twice as large as those based on F, and R- factors based on ALL data will be even larger.

### Fractional atomic coordinates and isotropic or equivalent isotropic displacement parameters (Å^2^)

**Table:**

	*x*	*y*	*z*	*U*_iso_*/*U*_eq_	
C1A	0.8230 (6)	0.7018 (5)	0.29229 (10)	0.0474 (13)	
C2A	0.9098 (7)	0.7759 (5)	0.27372 (11)	0.0595 (16)	
H2A	0.8808	0.8542	0.2721	0.071*	
C3A	1.0373 (7)	0.7347 (6)	0.25788 (12)	0.0692 (17)	
H3A	1.0956	0.7843	0.2458	0.083*	
C4A	1.0755 (7)	0.6195 (7)	0.26042 (13)	0.0676 (18)	
C5A	0.9969 (7)	0.5431 (5)	0.27796 (12)	0.0652 (16)	
H5A	1.0277	0.4651	0.2793	0.078*	
C6A	0.8699 (7)	0.5855 (5)	0.29361 (11)	0.0601 (15)	
H6A	0.8134	0.5343	0.3055	0.072*	
C7A	0.6928 (6)	0.7483 (5)	0.31027 (9)	0.0451 (12)	
C8A	0.6052 (6)	0.8464 (5)	0.30734 (11)	0.0554 (15)	
H8A	0.6133	0.8964	0.2914	0.066*	
C9A	0.4994 (6)	0.8657 (5)	0.33098 (12)	0.0557 (15)	
H9A	0.4306	0.9293	0.3318	0.067*	
C10A	0.5076 (6)	0.7834 (4)	0.35224 (10)	0.0421 (12)	
C11A	0.4200 (6)	0.7753 (4)	0.38050 (10)	0.0477 (13)	
H11A	0.3621	0.8478	0.3834	0.057*	
H11B	0.4969	0.7688	0.3962	0.057*	
C12A	0.3033 (5)	0.6737 (4)	0.38309 (9)	0.0369 (11)	
C13A	0.3383 (5)	0.5781 (4)	0.40050 (9)	0.0343 (11)	
H13A	0.4332	0.5779	0.4109	0.041*	
C14A	0.2385 (5)	0.4834 (4)	0.40311 (9)	0.0369 (11)	
C15A	0.0979 (5)	0.4846 (4)	0.38708 (10)	0.0404 (12)	
H15A	0.0283	0.4216	0.3883	0.049*	
C16A	0.0611 (6)	0.5791 (5)	0.36942 (10)	0.0482 (13)	
H16A	−0.0332	0.5784	0.3588	0.058*	
C17A	0.1607 (6)	0.6745 (5)	0.36711 (10)	0.0463 (12)	
C18A	0.1135 (7)	0.7778 (5)	0.34833 (12)	0.0690 (17)	
H18A	0.0041	0.7703	0.3428	0.104*	
H18B	0.1787	0.7798	0.3311	0.104*	
H18C	0.1278	0.8489	0.3592	0.104*	
C19A	0.2741 (5)	0.3786 (4)	0.42179 (9)	0.0357 (11)	
H19A	0.3865	0.3797	0.4273	0.043*	
C20A	0.1737 (5)	0.3719 (4)	0.44938 (9)	0.0326 (11)	
H20A	0.0615	0.3757	0.4438	0.039*	
C21A	0.2017 (5)	0.2584 (4)	0.46571 (8)	0.0339 (10)	
H21A	0.3096	0.2591	0.4737	0.041*	
C22A	0.1845 (5)	0.1543 (4)	0.44575 (9)	0.0340 (11)	
H22A	0.0726	0.1443	0.4405	0.041*	
C23A	0.2830 (6)	0.1693 (4)	0.41808 (9)	0.0424 (12)	
H23A	0.3968	0.1644	0.4223	0.051*	
C24A	0.2307 (7)	0.0731 (5)	0.39562 (10)	0.0588 (13)	
H24A	0.2386	−0.0039	0.4045	0.071*	
H24B	0.1215	0.0858	0.3897	0.071*	
C1B	1.2588 (6)	1.2409 (5)	0.30205 (11)	0.0562 (14)	
C2B	1.3509 (7)	1.1863 (6)	0.28096 (13)	0.0768 (19)	
H2B	1.3240	1.1110	0.2751	0.092*	
C3B	1.4806 (7)	1.2395 (8)	0.26844 (14)	0.085 (2)	
H3B	1.5430	1.2007	0.2548	0.102*	
C4B	1.5136 (8)	1.3496 (8)	0.27672 (16)	0.085 (2)	
C5B	1.4341 (8)	1.4082 (7)	0.29795 (16)	0.086 (2)	
H5B	1.4656	1.4824	0.3039	0.103*	
C6B	1.3035 (7)	1.3528 (6)	0.31044 (13)	0.0697 (17)	
H6B	1.2451	1.3917	0.3247	0.084*	
C7B	1.1148 (6)	1.1843 (5)	0.31376 (10)	0.0499 (13)	
C8B	1.0747 (7)	1.0717 (6)	0.31352 (12)	0.0637 (16)	
H8B	1.1397	1.0133	0.3060	0.076*	
C9B	0.9242 (7)	1.0495 (5)	0.32585 (12)	0.0627 (16)	
H9B	0.8806	0.9750	0.3274	0.075*	
C10B	0.8499 (6)	1.1468 (5)	0.33522 (9)	0.0441 (13)	
C11B	0.6910 (6)	1.1544 (5)	0.35001 (10)	0.0532 (14)	
H11C	0.6363	1.0801	0.3480	0.064*	
H11D	0.6274	1.2139	0.3404	0.064*	
C12B	0.7072 (5)	1.1844 (5)	0.38244 (9)	0.0389 (12)	
C13B	0.7355 (5)	1.0932 (4)	0.40164 (9)	0.0355 (11)	
H13B	0.7394	1.0170	0.3945	0.043*	
C14B	0.7583 (5)	1.1132 (4)	0.43165 (9)	0.0334 (11)	
C15B	0.7535 (5)	1.2272 (4)	0.44152 (10)	0.0373 (11)	
H15B	0.7706	1.2431	0.4612	0.045*	
C16B	0.7237 (5)	1.3172 (4)	0.42246 (10)	0.0437 (12)	
H16B	0.7195	1.3933	0.4296	0.052*	
C17B	0.6997 (5)	1.2985 (4)	0.39290 (11)	0.0411 (12)	
C18B	0.6624 (6)	1.4007 (5)	0.37319 (11)	0.0586 (15)	
H18D	0.6825	1.4725	0.3835	0.088*	
H18E	0.5528	1.3977	0.3675	0.088*	
H18F	0.7281	1.3971	0.3561	0.088*	
C19B	0.7807 (5)	1.0121 (4)	0.45231 (9)	0.0330 (10)	
H19B	0.8318	1.0399	0.4703	0.040*	
C20B	0.6217 (5)	0.9556 (4)	0.45993 (9)	0.0302 (10)	
H20B	0.5595	0.9465	0.4420	0.036*	
C21B	0.6378 (5)	0.8365 (4)	0.47464 (9)	0.0353 (11)	
H21B	0.6707	0.8489	0.4950	0.042*	
C22B	0.7590 (5)	0.7601 (4)	0.46008 (9)	0.0341 (10)	
H22B	0.7200	0.7347	0.4409	0.041*	
C23B	0.9139 (5)	0.8280 (4)	0.45649 (9)	0.0335 (11)	
H23B	0.9519	0.8539	0.4757	0.040*	
C24B	1.0424 (5)	0.7610 (4)	0.44114 (10)	0.0466 (12)	
H24C	1.0593	0.6864	0.4508	0.056*	
H24D	1.0119	0.7461	0.4210	0.056*	
F1A	1.2033 (4)	0.5781 (4)	0.24514 (8)	0.0935 (12)	
F1B	1.6394 (5)	1.4058 (5)	0.26352 (10)	0.1351 (18)	
O1A	0.2438 (4)	0.2775 (3)	0.40429 (6)	0.0421 (8)	
O2A	0.2095 (4)	0.4714 (3)	0.46711 (6)	0.0440 (8)	
H2A1	0.2400	0.4495	0.4832	0.066*	
O3A	0.0913 (4)	0.2435 (3)	0.48929 (6)	0.0441 (8)	
H3A1	0.0983	0.2991	0.5006	0.066*	
O4A	0.2376 (4)	0.0535 (3)	0.46192 (7)	0.0406 (8)	
H4A	0.1973	−0.0057	0.4550	0.061*	
O5A	0.3311 (6)	0.0803 (4)	0.37124 (10)	0.0913 (14)	
H5A1	0.3019	0.1340	0.3606	0.137*	
O1B	0.8812 (3)	0.9285 (3)	0.43833 (6)	0.0370 (7)	
O2B	0.5393 (3)	1.0343 (3)	0.47925 (6)	0.0371 (8)	
H2B1	0.4465	1.0401	0.4741	0.056*	
O3B	0.4879 (4)	0.7782 (3)	0.47478 (7)	0.0472 (8)	
H3B1	0.4316	0.8062	0.4876	0.071*	
O4B	0.7951 (4)	0.6600 (3)	0.47784 (7)	0.0463 (8)	
H4B	0.7223	0.6126	0.4768	0.069*	
O5B	1.1853 (4)	0.8292 (3)	0.44195 (8)	0.0559 (9)	
H5B1	1.2601	0.7872	0.4466	0.084*	
O6	0.5619 (4)	0.4920 (3)	0.47940 (8)	0.0602 (10)	
H61	0.4711	0.4818	0.4733	0.090*	
H62	0.5714	0.4204	0.4819	0.090*	
S1A	0.64414 (16)	0.67821 (12)	0.34263 (3)	0.0523 (4)	
S1B	0.96635 (17)	1.26679 (13)	0.32902 (3)	0.0598 (4)	

### Atomic displacement parameters (Å^2^)

**Table:**

	*U*^11^	*U*^22^	*U*^33^	*U*^12^	*U*^13^	*U*^23^
C1A	0.055 (3)	0.047 (4)	0.041 (3)	−0.002 (3)	−0.004 (3)	0.002 (2)
C2A	0.067 (4)	0.057 (4)	0.054 (3)	−0.010 (3)	−0.002 (3)	0.010 (3)
C3A	0.063 (4)	0.085 (5)	0.060 (4)	0.000 (4)	0.014 (3)	0.011 (4)
C4A	0.060 (4)	0.083 (5)	0.060 (4)	−0.005 (4)	0.005 (3)	−0.011 (4)
C5A	0.077 (4)	0.057 (4)	0.062 (4)	0.005 (4)	0.010 (4)	−0.002 (3)
C6A	0.071 (4)	0.049 (4)	0.060 (3)	−0.009 (3)	0.009 (3)	0.006 (3)
C7A	0.052 (3)	0.039 (3)	0.044 (3)	−0.014 (3)	0.000 (2)	0.013 (3)
C8A	0.066 (4)	0.045 (4)	0.055 (3)	0.006 (3)	0.010 (3)	0.017 (3)
C9A	0.062 (4)	0.035 (3)	0.070 (4)	0.007 (3)	0.003 (3)	0.009 (3)
C10A	0.047 (3)	0.032 (3)	0.048 (3)	0.005 (2)	0.006 (2)	0.008 (2)
C11A	0.061 (3)	0.030 (3)	0.052 (3)	−0.002 (3)	0.005 (3)	0.000 (2)
C12A	0.047 (3)	0.025 (3)	0.038 (2)	−0.004 (2)	0.008 (2)	−0.005 (2)
C13A	0.033 (3)	0.035 (3)	0.035 (2)	−0.001 (2)	0.001 (2)	−0.004 (2)
C14A	0.042 (3)	0.033 (3)	0.036 (2)	0.002 (2)	0.004 (2)	0.000 (2)
C15A	0.042 (3)	0.031 (3)	0.048 (3)	−0.003 (2)	−0.009 (2)	0.003 (2)
C16A	0.045 (3)	0.047 (3)	0.053 (3)	−0.004 (3)	−0.013 (3)	0.010 (3)
C17A	0.055 (3)	0.042 (3)	0.042 (3)	0.002 (3)	−0.005 (3)	0.005 (2)
C18A	0.074 (4)	0.050 (4)	0.083 (4)	−0.001 (3)	−0.020 (3)	0.021 (3)
C19A	0.034 (3)	0.029 (3)	0.044 (3)	0.000 (2)	0.002 (2)	0.002 (2)
C20A	0.037 (3)	0.023 (3)	0.038 (2)	−0.002 (2)	0.000 (2)	−0.001 (2)
C21A	0.033 (2)	0.033 (3)	0.036 (2)	−0.003 (2)	−0.004 (2)	0.003 (2)
C22A	0.033 (2)	0.024 (3)	0.045 (3)	0.002 (2)	−0.005 (2)	0.002 (2)
C23A	0.049 (3)	0.037 (3)	0.042 (3)	0.010 (3)	−0.003 (2)	−0.002 (2)
C24A	0.086	0.044 (3)	0.046 (3)	0.026 (3)	0.038 (3)	0.008 (3)
C1B	0.058 (3)	0.063 (4)	0.047 (3)	0.017 (3)	−0.003 (3)	0.002 (3)
C2B	0.062 (4)	0.089 (5)	0.079 (4)	0.006 (4)	0.001 (4)	−0.013 (4)
C3B	0.056 (4)	0.116 (7)	0.082 (5)	0.004 (5)	0.012 (4)	−0.016 (5)
C4B	0.051 (4)	0.116 (7)	0.088 (5)	−0.020 (4)	0.008 (4)	0.000 (5)
C5B	0.070 (5)	0.090 (6)	0.098 (5)	−0.013 (4)	0.001 (4)	−0.001 (5)
C6B	0.064 (4)	0.078 (5)	0.068 (4)	0.006 (4)	0.009 (3)	−0.007 (3)
C7B	0.048 (3)	0.056 (4)	0.046 (3)	0.008 (3)	0.003 (3)	−0.006 (3)
C8B	0.062 (4)	0.056 (4)	0.073 (4)	0.016 (3)	0.002 (3)	−0.003 (3)
C9B	0.075 (4)	0.049 (4)	0.064 (4)	−0.006 (3)	−0.012 (3)	0.004 (3)
C10B	0.058 (3)	0.043 (3)	0.032 (3)	−0.014 (3)	0.000 (2)	0.009 (2)
C11B	0.057 (3)	0.058 (4)	0.045 (3)	−0.006 (3)	−0.002 (3)	0.011 (3)
C12B	0.031 (2)	0.051 (3)	0.035 (2)	−0.003 (2)	−0.004 (2)	0.007 (2)
C13B	0.040 (3)	0.033 (3)	0.034 (2)	−0.002 (2)	0.002 (2)	0.005 (2)
C14B	0.028 (2)	0.029 (3)	0.043 (3)	0.003 (2)	0.003 (2)	0.009 (2)
C15B	0.036 (2)	0.034 (3)	0.042 (2)	0.002 (2)	0.004 (2)	0.006 (2)
C16B	0.040 (3)	0.032 (3)	0.059 (3)	0.002 (2)	0.013 (3)	0.008 (3)
C17B	0.033 (3)	0.038 (3)	0.052 (3)	0.006 (2)	0.007 (2)	0.018 (2)
C18B	0.055 (3)	0.050 (4)	0.070 (4)	0.014 (3)	0.005 (3)	0.026 (3)
C19B	0.037 (3)	0.028 (3)	0.034 (2)	0.006 (2)	0.001 (2)	0.002 (2)
C20B	0.030 (2)	0.031 (3)	0.029 (2)	0.001 (2)	0.000 (2)	−0.0001 (19)
C21B	0.034 (2)	0.034 (3)	0.038 (2)	−0.005 (2)	0.000 (2)	0.004 (2)
C22B	0.038 (3)	0.023 (3)	0.041 (2)	−0.002 (2)	−0.007 (2)	0.005 (2)
C23B	0.040 (3)	0.022 (2)	0.038 (2)	0.011 (2)	0.000 (2)	−0.001 (2)
C24B	0.045 (3)	0.038 (3)	0.056 (3)	0.004 (3)	0.003 (2)	0.006 (3)
F1A	0.074 (2)	0.115 (3)	0.091 (2)	0.010 (2)	0.028 (2)	−0.011 (2)
F1B	0.076 (3)	0.183 (5)	0.146 (4)	−0.037 (3)	0.029 (3)	−0.007 (4)
O1A	0.056 (2)	0.0316 (19)	0.0392 (16)	0.0070 (17)	0.0008 (15)	0.0012 (15)
O2A	0.061 (2)	0.031 (2)	0.0395 (17)	−0.0058 (17)	−0.0058 (17)	−0.0042 (15)
O3A	0.057 (2)	0.039 (2)	0.0357 (17)	−0.0041 (18)	0.0126 (15)	0.0011 (16)
O4A	0.045 (2)	0.0237 (18)	0.0527 (19)	−0.0026 (16)	−0.0045 (16)	0.0017 (15)
O5A	0.101 (4)	0.085 (4)	0.088 (3)	0.020 (3)	0.006 (3)	−0.006 (3)
O1B	0.0411 (17)	0.0312 (19)	0.0388 (16)	0.0069 (15)	0.0085 (15)	0.0103 (15)
O2B	0.0341 (16)	0.037 (2)	0.0405 (17)	0.0026 (15)	0.0010 (15)	−0.0046 (15)
O3B	0.0437 (19)	0.040 (2)	0.058 (2)	−0.0110 (17)	0.0070 (16)	0.0015 (16)
O4B	0.047 (2)	0.0264 (19)	0.066 (2)	−0.0040 (16)	−0.0110 (18)	0.0141 (17)
O5B	0.042 (2)	0.042 (2)	0.084 (3)	0.0068 (18)	0.009 (2)	−0.002 (2)
O6	0.062 (2)	0.041 (2)	0.079 (3)	−0.0114 (19)	−0.011 (2)	0.007 (2)
S1A	0.0602 (8)	0.0419 (8)	0.0549 (8)	0.0073 (7)	0.0109 (7)	0.0161 (7)
S1B	0.0673 (9)	0.0499 (10)	0.0621 (8)	−0.0040 (8)	0.0172 (7)	−0.0030 (7)

### Geometric parameters (Å, º)

**Table:**

C1A—C6A	1.387 (7)	C3B—C4B	1.344 (9)
C1A—C2A	1.405 (7)	C3B—H3B	0.9300
C1A—C7A	1.470 (7)	C4B—C5B	1.356 (9)
C2A—C3A	1.378 (8)	C4B—F1B	1.378 (7)
C2A—H2A	0.9300	C5B—C6B	1.391 (8)
C3A—C4A	1.360 (8)	C5B—H5B	0.9300
C3A—H3A	0.9300	C6B—H6B	0.9300
C4A—C5A	1.358 (8)	C7B—C8B	1.330 (8)
C4A—F1A	1.368 (7)	C7B—S1B	1.715 (5)
C5A—C6A	1.376 (8)	C8B—C9B	1.411 (8)
C5A—H5A	0.9300	C8B—H8B	0.9300
C6A—H6A	0.9300	C9B—C10B	1.345 (7)
C7A—C8A	1.348 (7)	C9B—H9B	0.9300
C7A—S1A	1.731 (4)	C10B—C11B	1.502 (7)
C8A—C9A	1.418 (7)	C10B—S1B	1.710 (5)
C8A—H8A	0.9300	C11B—C12B	1.527 (6)
C9A—C10A	1.354 (6)	C11B—H11C	0.9700
C9A—H9A	0.9300	C11B—H11D	0.9700
C10A—C11A	1.491 (6)	C12B—C13B	1.383 (6)
C10A—S1A	1.721 (5)	C12B—C17B	1.390 (6)
C11A—C12A	1.526 (6)	C13B—C14B	1.404 (6)
C11A—H11A	0.9700	C13B—H13B	0.9300
C11A—H11B	0.9700	C14B—C15B	1.379 (6)
C12A—C13A	1.383 (6)	C14B—C19B	1.504 (6)
C12A—C17A	1.407 (6)	C15B—C16B	1.371 (6)
C13A—C14A	1.375 (6)	C15B—H15B	0.9300
C13A—H13A	0.9300	C16B—C17B	1.383 (6)
C14A—C15A	1.394 (6)	C16B—H16B	0.9300
C14A—C19A	1.501 (6)	C17B—C18B	1.508 (6)
C15A—C16A	1.383 (6)	C18B—H18D	0.9600
C15A—H15A	0.9300	C18B—H18E	0.9600
C16A—C17A	1.379 (7)	C18B—H18F	0.9600
C16A—H16A	0.9300	C19B—O1B	1.427 (5)
C17A—C18A	1.513 (7)	C19B—C20B	1.528 (6)
C18A—H18A	0.9600	C19B—H19B	0.9800
C18A—H18B	0.9600	C20B—O2B	1.439 (5)
C18A—H18C	0.9600	C20B—C21B	1.524 (6)
C19A—O1A	1.428 (5)	C20B—H20B	0.9800
C19A—C20A	1.521 (6)	C21B—O3B	1.428 (5)
C19A—H19A	0.9800	C21B—C22B	1.499 (6)
C20A—O2A	1.428 (5)	C21B—H21B	0.9800
C20A—C21A	1.515 (6)	C22B—O4B	1.436 (5)
C20A—H20A	0.9800	C22B—C23B	1.526 (6)
C21A—O3A	1.434 (5)	C22B—H22B	0.9800
C21A—C22A	1.506 (6)	C23B—O1B	1.444 (5)
C21A—H21A	0.9800	C23B—C24B	1.500 (6)
C22A—O4A	1.439 (5)	C23B—H23B	0.9800
C22A—C23A	1.522 (6)	C24B—O5B	1.435 (5)
C22A—H22A	0.9800	C24B—H24C	0.9700
C23A—O1A	1.427 (5)	C24B—H24D	0.9700
C23A—C24A	1.567 (7)	O2A—H2A1	0.8200
C23A—H23A	0.9800	O3A—H3A1	0.8200
C24A—O5A	1.401 (6)	O4A—H4A	0.8200
C24A—H24A	0.9700	O5A—H5A1	0.8200
C24A—H24B	0.9700	O2B—H2B1	0.8200
C1B—C2B	1.386 (7)	O3B—H3B1	0.8200
C1B—C6B	1.387 (8)	O4B—H4B	0.8200
C1B—C7B	1.476 (7)	O5B—H5B1	0.8200
C2B—C3B	1.375 (8)	O6—H61	0.8228
C2B—H2B	0.9300	O6—H62	0.8292
			
C6A—C1A—C2A	117.1 (5)	C4B—C3B—H3B	121.3
C6A—C1A—C7A	122.3 (5)	C2B—C3B—H3B	121.3
C2A—C1A—C7A	120.5 (5)	C3B—C4B—C5B	124.1 (7)
C3A—C2A—C1A	121.1 (6)	C3B—C4B—F1B	118.2 (7)
C3A—C2A—H2A	119.4	C5B—C4B—F1B	117.7 (8)
C1A—C2A—H2A	119.4	C4B—C5B—C6B	117.4 (7)
C4A—C3A—C2A	118.0 (6)	C4B—C5B—H5B	121.3
C4A—C3A—H3A	121.0	C6B—C5B—H5B	121.3
C2A—C3A—H3A	121.0	C1B—C6B—C5B	121.3 (6)
C5A—C4A—C3A	123.9 (6)	C1B—C6B—H6B	119.3
C5A—C4A—F1A	117.5 (6)	C5B—C6B—H6B	119.3
C3A—C4A—F1A	118.5 (6)	C8B—C7B—C1B	129.0 (5)
C4A—C5A—C6A	117.4 (6)	C8B—C7B—S1B	110.5 (4)
C4A—C5A—H5A	121.3	C1B—C7B—S1B	120.4 (4)
C6A—C5A—H5A	121.3	C7B—C8B—C9B	113.5 (6)
C5A—C6A—C1A	122.4 (5)	C7B—C8B—H8B	123.3
C5A—C6A—H6A	118.8	C9B—C8B—H8B	123.3
C1A—C6A—H6A	118.8	C10B—C9B—C8B	113.4 (5)
C8A—C7A—C1A	130.8 (4)	C10B—C9B—H9B	123.3
C8A—C7A—S1A	109.9 (4)	C8B—C9B—H9B	123.3
C1A—C7A—S1A	119.1 (4)	C9B—C10B—C11B	127.3 (5)
C7A—C8A—C9A	113.5 (5)	C9B—C10B—S1B	110.0 (4)
C7A—C8A—H8A	123.3	C11B—C10B—S1B	122.6 (4)
C9A—C8A—H8A	123.3	C10B—C11B—C12B	111.7 (4)
C10A—C9A—C8A	114.0 (5)	C10B—C11B—H11C	109.3
C10A—C9A—H9A	123.0	C12B—C11B—H11C	109.3
C8A—C9A—H9A	123.0	C10B—C11B—H11D	109.3
C9A—C10A—C11A	129.8 (5)	C12B—C11B—H11D	109.3
C9A—C10A—S1A	109.6 (4)	H11C—C11B—H11D	107.9
C11A—C10A—S1A	120.6 (3)	C13B—C12B—C17B	119.8 (4)
C10A—C11A—C12A	115.7 (4)	C13B—C12B—C11B	117.5 (5)
C10A—C11A—H11A	108.4	C17B—C12B—C11B	122.7 (4)
C12A—C11A—H11A	108.4	C12B—C13B—C14B	121.4 (4)
C10A—C11A—H11B	108.4	C12B—C13B—H13B	119.3
C12A—C11A—H11B	108.4	C14B—C13B—H13B	119.3
H11A—C11A—H11B	107.4	C15B—C14B—C13B	118.0 (4)
C13A—C12A—C17A	119.1 (4)	C15B—C14B—C19B	121.6 (4)
C13A—C12A—C11A	120.6 (4)	C13B—C14B—C19B	120.4 (4)
C17A—C12A—C11A	120.3 (4)	C16B—C15B—C14B	120.4 (4)
C14A—C13A—C12A	122.8 (4)	C16B—C15B—H15B	119.8
C14A—C13A—H13A	118.6	C14B—C15B—H15B	119.8
C12A—C13A—H13A	118.6	C15B—C16B—C17B	122.1 (5)
C13A—C14A—C15A	117.8 (4)	C15B—C16B—H16B	118.9
C13A—C14A—C19A	123.7 (4)	C17B—C16B—H16B	118.9
C15A—C14A—C19A	118.5 (4)	C16B—C17B—C12B	118.3 (4)
C16A—C15A—C14A	120.3 (5)	C16B—C17B—C18B	119.6 (5)
C16A—C15A—H15A	119.8	C12B—C17B—C18B	122.0 (5)
C14A—C15A—H15A	119.8	C17B—C18B—H18D	109.5
C17A—C16A—C15A	121.7 (5)	C17B—C18B—H18E	109.5
C17A—C16A—H16A	119.2	H18D—C18B—H18E	109.5
C15A—C16A—H16A	119.2	C17B—C18B—H18F	109.5
C16A—C17A—C12A	118.4 (5)	H18D—C18B—H18F	109.5
C16A—C17A—C18A	120.0 (4)	H18E—C18B—H18F	109.5
C12A—C17A—C18A	121.6 (5)	O1B—C19B—C14B	107.9 (3)
C17A—C18A—H18A	109.5	O1B—C19B—C20B	109.9 (3)
C17A—C18A—H18B	109.5	C14B—C19B—C20B	110.9 (4)
H18A—C18A—H18B	109.5	O1B—C19B—H19B	109.4
C17A—C18A—H18C	109.5	C14B—C19B—H19B	109.4
H18A—C18A—H18C	109.5	C20B—C19B—H19B	109.4
H18B—C18A—H18C	109.5	O2B—C20B—C21B	109.3 (3)
O1A—C19A—C14A	106.9 (3)	O2B—C20B—C19B	107.4 (3)
O1A—C19A—C20A	109.0 (3)	C21B—C20B—C19B	113.5 (3)
C14A—C19A—C20A	113.6 (4)	O2B—C20B—H20B	108.8
O1A—C19A—H19A	109.1	C21B—C20B—H20B	108.8
C14A—C19A—H19A	109.1	C19B—C20B—H20B	108.8
C20A—C19A—H19A	109.1	O3B—C21B—C22B	109.5 (4)
O2A—C20A—C21A	111.7 (3)	O3B—C21B—C20B	109.8 (4)
O2A—C20A—C19A	108.2 (3)	C22B—C21B—C20B	112.6 (3)
C21A—C20A—C19A	111.4 (4)	O3B—C21B—H21B	108.3
O2A—C20A—H20A	108.5	C22B—C21B—H21B	108.3
C21A—C20A—H20A	108.5	C20B—C21B—H21B	108.3
C19A—C20A—H20A	108.5	O4B—C22B—C21B	110.9 (3)
O3A—C21A—C22A	107.4 (4)	O4B—C22B—C23B	106.5 (3)
O3A—C21A—C20A	111.7 (4)	C21B—C22B—C23B	109.5 (4)
C22A—C21A—C20A	111.3 (3)	O4B—C22B—H22B	109.9
O3A—C21A—H21A	108.8	C21B—C22B—H22B	109.9
C22A—C21A—H21A	108.8	C23B—C22B—H22B	109.9
C20A—C21A—H21A	108.8	O1B—C23B—C24B	105.9 (3)
O4A—C22A—C21A	106.9 (3)	O1B—C23B—C22B	107.6 (3)
O4A—C22A—C23A	110.3 (4)	C24B—C23B—C22B	114.1 (4)
C21A—C22A—C23A	111.2 (4)	O1B—C23B—H23B	109.7
O4A—C22A—H22A	109.5	C24B—C23B—H23B	109.7
C21A—C22A—H22A	109.5	C22B—C23B—H23B	109.7
C23A—C22A—H22A	109.5	O5B—C24B—C23B	108.5 (4)
O1A—C23A—C22A	109.8 (4)	O5B—C24B—H24C	110.0
O1A—C23A—C24A	104.7 (3)	C23B—C24B—H24C	110.0
C22A—C23A—C24A	108.2 (4)	O5B—C24B—H24D	110.0
O1A—C23A—H23A	111.3	C23B—C24B—H24D	110.0
C22A—C23A—H23A	111.3	H24C—C24B—H24D	108.4
C24A—C23A—H23A	111.3	C23A—O1A—C19A	114.3 (3)
O5A—C24A—C23A	108.1 (5)	C20A—O2A—H2A1	109.5
O5A—C24A—H24A	110.1	C21A—O3A—H3A1	109.5
C23A—C24A—H24A	110.1	C22A—O4A—H4A	109.5
O5A—C24A—H24B	110.1	C24A—O5A—H5A1	109.5
C23A—C24A—H24B	110.1	C19B—O1B—C23B	112.8 (3)
H24A—C24A—H24B	108.4	C20B—O2B—H2B1	109.5
C2B—C1B—C6B	117.1 (6)	C21B—O3B—H3B1	109.5
C2B—C1B—C7B	121.0 (6)	C22B—O4B—H4B	109.5
C6B—C1B—C7B	121.8 (5)	C24B—O5B—H5B1	109.5
C3B—C2B—C1B	122.4 (6)	H61—O6—H62	89.8
C3B—C2B—H2B	118.8	C10A—S1A—C7A	93.0 (2)
C1B—C2B—H2B	118.8	C10B—S1B—C7B	92.6 (3)
C4B—C3B—C2B	117.5 (6)		
			
C6A—C1A—C2A—C3A	−0.9 (8)	C7B—C1B—C6B—C5B	−176.1 (5)
C7A—C1A—C2A—C3A	176.6 (5)	C4B—C5B—C6B—C1B	1.6 (10)
C1A—C2A—C3A—C4A	0.9 (8)	C2B—C1B—C7B—C8B	20.5 (9)
C2A—C3A—C4A—C5A	−1.0 (10)	C6B—C1B—C7B—C8B	−162.9 (6)
C2A—C3A—C4A—F1A	−179.0 (5)	C2B—C1B—C7B—S1B	−156.6 (4)
C3A—C4A—C5A—C6A	1.1 (9)	C6B—C1B—C7B—S1B	20.1 (7)
F1A—C4A—C5A—C6A	179.1 (5)	C1B—C7B—C8B—C9B	−177.8 (5)
C4A—C5A—C6A—C1A	−1.0 (9)	S1B—C7B—C8B—C9B	−0.5 (6)
C2A—C1A—C6A—C5A	0.9 (8)	C7B—C8B—C9B—C10B	0.6 (7)
C7A—C1A—C6A—C5A	−176.5 (5)	C8B—C9B—C10B—C11B	−178.3 (5)
C6A—C1A—C7A—C8A	−161.5 (6)	C8B—C9B—C10B—S1B	−0.3 (6)
C2A—C1A—C7A—C8A	21.2 (8)	C9B—C10B—C11B—C12B	108.0 (6)
C6A—C1A—C7A—S1A	24.2 (6)	S1B—C10B—C11B—C12B	−69.8 (5)
C2A—C1A—C7A—S1A	−153.1 (4)	C10B—C11B—C12B—C13B	−84.3 (5)
C1A—C7A—C8A—C9A	−174.5 (5)	C10B—C11B—C12B—C17B	93.7 (6)
S1A—C7A—C8A—C9A	0.2 (6)	C17B—C12B—C13B—C14B	−0.6 (7)
C7A—C8A—C9A—C10A	0.9 (7)	C11B—C12B—C13B—C14B	177.4 (4)
C8A—C9A—C10A—C11A	178.1 (5)	C12B—C13B—C14B—C15B	−0.7 (6)
C8A—C9A—C10A—S1A	−1.5 (6)	C12B—C13B—C14B—C19B	177.0 (4)
C9A—C10A—C11A—C12A	113.3 (6)	C13B—C14B—C15B—C16B	1.4 (6)
S1A—C10A—C11A—C12A	−67.1 (5)	C19B—C14B—C15B—C16B	−176.2 (4)
C10A—C11A—C12A—C13A	106.6 (5)	C14B—C15B—C16B—C17B	−0.9 (7)
C10A—C11A—C12A—C17A	−71.5 (6)	C15B—C16B—C17B—C12B	−0.4 (7)
C17A—C12A—C13A—C14A	−0.6 (7)	C15B—C16B—C17B—C18B	178.1 (4)
C11A—C12A—C13A—C14A	−178.7 (4)	C13B—C12B—C17B—C16B	1.1 (7)
C12A—C13A—C14A—C15A	0.9 (7)	C11B—C12B—C17B—C16B	−176.8 (4)
C12A—C13A—C14A—C19A	179.8 (4)	C13B—C12B—C17B—C18B	−177.3 (4)
C13A—C14A—C15A—C16A	−0.5 (7)	C11B—C12B—C17B—C18B	4.7 (7)
C19A—C14A—C15A—C16A	−179.5 (4)	C15B—C14B—C19B—O1B	−141.6 (4)
C14A—C15A—C16A—C17A	−0.2 (8)	C13B—C14B—C19B—O1B	40.9 (5)
C15A—C16A—C17A—C12A	0.5 (8)	C15B—C14B—C19B—C20B	98.1 (5)
C15A—C16A—C17A—C18A	−178.0 (5)	C13B—C14B—C19B—C20B	−79.5 (5)
C13A—C12A—C17A—C16A	−0.1 (7)	O1B—C19B—C20B—O2B	167.9 (3)
C11A—C12A—C17A—C16A	178.0 (4)	C14B—C19B—C20B—O2B	−72.9 (4)
C13A—C12A—C17A—C18A	178.3 (4)	O1B—C19B—C20B—C21B	46.9 (5)
C11A—C12A—C17A—C18A	−3.6 (7)	C14B—C19B—C20B—C21B	166.2 (4)
C13A—C14A—C19A—O1A	−132.3 (4)	O2B—C20B—C21B—O3B	73.6 (4)
C15A—C14A—C19A—O1A	46.7 (5)	C19B—C20B—C21B—O3B	−166.5 (3)
C13A—C14A—C19A—C20A	107.5 (5)	O2B—C20B—C21B—C22B	−164.1 (3)
C15A—C14A—C19A—C20A	−73.6 (5)	C19B—C20B—C21B—C22B	−44.2 (5)
O1A—C19A—C20A—O2A	178.0 (3)	O3B—C21B—C22B—O4B	−69.7 (4)
C14A—C19A—C20A—O2A	−62.9 (5)	C20B—C21B—C22B—O4B	167.8 (3)
O1A—C19A—C20A—C21A	54.8 (5)	O3B—C21B—C22B—C23B	173.0 (3)
C14A—C19A—C20A—C21A	173.9 (4)	C20B—C21B—C22B—C23B	50.5 (5)
O2A—C20A—C21A—O3A	67.4 (5)	O4B—C22B—C23B—O1B	179.2 (3)
C19A—C20A—C21A—O3A	−171.4 (3)	C21B—C22B—C23B—O1B	−60.8 (4)
O2A—C20A—C21A—C22A	−172.5 (4)	O4B—C22B—C23B—C24B	62.0 (5)
C19A—C20A—C21A—C22A	−51.4 (5)	C21B—C22B—C23B—C24B	−178.0 (4)
O3A—C21A—C22A—O4A	−66.4 (4)	O1B—C23B—C24B—O5B	67.8 (4)
C20A—C21A—C22A—O4A	171.0 (4)	C22B—C23B—C24B—O5B	−174.0 (4)
O3A—C21A—C22A—C23A	173.2 (3)	C22A—C23A—O1A—C19A	61.0 (5)
C20A—C21A—C22A—C23A	50.5 (5)	C24A—C23A—O1A—C19A	176.9 (4)
O4A—C22A—C23A—O1A	−172.3 (3)	C14A—C19A—O1A—C23A	175.6 (4)
C21A—C22A—C23A—O1A	−53.9 (5)	C20A—C19A—O1A—C23A	−61.2 (5)
O4A—C22A—C23A—C24A	74.0 (4)	C14B—C19B—O1B—C23B	178.8 (3)
C21A—C22A—C23A—C24A	−167.6 (4)	C20B—C19B—O1B—C23B	−60.1 (4)
O1A—C23A—C24A—O5A	68.2 (5)	C24B—C23B—O1B—C19B	−169.8 (3)
C22A—C23A—C24A—O5A	−174.7 (4)	C22B—C23B—O1B—C19B	67.7 (4)
C6B—C1B—C2B—C3B	−0.5 (9)	C9A—C10A—S1A—C7A	1.4 (4)
C7B—C1B—C2B—C3B	176.3 (5)	C11A—C10A—S1A—C7A	−178.3 (4)
C1B—C2B—C3B—C4B	−2.0 (10)	C8A—C7A—S1A—C10A	−0.9 (4)
C2B—C3B—C4B—C5B	4.7 (11)	C1A—C7A—S1A—C10A	174.5 (4)
C2B—C3B—C4B—F1B	−177.5 (6)	C9B—C10B—S1B—C7B	0.0 (4)
C3B—C4B—C5B—C6B	−4.5 (11)	C11B—C10B—S1B—C7B	178.1 (4)
F1B—C4B—C5B—C6B	177.7 (6)	C8B—C7B—S1B—C10B	0.3 (4)
C2B—C1B—C6B—C5B	0.7 (8)	C1B—C7B—S1B—C10B	177.8 (4)

### Hydrogen-bond geometry (Å, º)

**Table:**

*D*—H···*A*	*D*—H	H···*A*	*D*···*A*	*D*—H···*A*
O2*A*—H2*A*1···O2*B*^i^	0.82	2.42	2.841 (4)	113
O3*A*—H3*A*1···O2*B*^i^	0.82	2.17	2.951 (4)	158
O4*A*—H4*A*···O5*B*^ii^	0.82	1.98	2.756 (5)	157
O2*B*—H2*B*1···O4*A*^iii^	0.82	1.85	2.672 (4)	179
O3*B*—H3*B*1···O4*B*^i^	0.82	1.99	2.797 (4)	168
O4*B*—H4*B*···O6	0.82	1.93	2.749 (5)	172
O5*B*—H5*B*1···O3*B*^iv^	0.82	2.31	3.015 (5)	144
O6—H61···O2*A*	0.82	2.23	3.031 (5)	166
O6—H62···O3*A*^v^	0.83	2.30	3.058 (5)	153

Symmetry codes: (i) *x*−1/2, −*y*+3/2, −*z*+1; (ii) *x*−1, *y*−1, *z*; (iii) *x*, *y*+1, *z*; (iv) *x*+1, *y*, *z*; (v) *x*+1/2, −*y*+1/2, −*z*+1.
